# Acquired Hemophilia A Treated with Recombinant Porcine Factor VIII: Case Report and Literature Review on Its Efficacy

**DOI:** 10.3390/hematolrep15010003

**Published:** 2023-01-06

**Authors:** Matteo Borro, Riccardo Tassara, Luca Paris, Nathan Artom, Marcello Brignone, Lara Rebella, Rodolfo Tassara

**Affiliations:** 1 Department of Internal Medicine, San Paolo Hospital, Via Genova 30, 17100 Savona, Italy; 2 Department of Internal Medicine, University of Genoa and Policlinico San Martino, Viale Benedetto XV, 6, 16132 Genova, Italy

**Keywords:** Acquired Hemophilia A, susoctocog-alfa, prolonged activated partial thromboplastin time

## Abstract

Acquired hemophilia A (AHA) is a bleeding disorder due to the presence of neutralizing autoantibodies named inhibitors in patients with a previously normal hemostasis. Recent international recommendations suggest the use of bypassing agents or substitutive therapy as the first-line treatment, usually preferring the former. The adequate hemostatic therapy needs an accurate balance between bleeding and thrombotic risks. We report a clinical case of acquired hemophilia A successfully treated with recombinant porcine factor VIII (Susoctocog alfa) as the first-line treatment. Despite the patient having a high-risk thrombotic score and a history of recent myocardial infarction, our experience showed the absence of thrombotic complications related to the use of Susoctocog alfa and a complete restoration of hemostatic parameters. Limited literature is present on the use of recombinant porcine factor VIII as a first-line treatment, and our report supports its use, especially when the thrombotic risk is high.

## 1. Introduction

Acquired hemophilia A (AHA) is a rare bleeding disorder in patients with a previously normal hemostasis characterized by the presence of neutralizing autoantibodies named inhibitors, whose target is the coagulation factor VIII (FVIII) [1]. Typically, the incidence of AHA is associated with pregnancy and older age, e.g., over 60 years old [2]. Over 50% of patients with AHA have comorbidities, frequently autoimmune disorders or malignancy [2,3]. The clinical manifestations may range from asymptomatic to life-threatening bleeding, but subcutaneous hematomas are present in 80% of patients and frequently the first sign of the disease [4]. Moreover, especially in the elderly with multiple chronic diseases, the patient’s ongoing medications, i.e., antiplatelet agents and anticoagulants, may interfere with the clinical picture and delay the correct diagnosis [1]. Laboratory investigations lead the diagnostic pathway of AHA and are characterized by prolonged activated partial thromboplastin time (APTT) confirmed with the APTT mixing study, various levels of reduced FVIII activity (usually ranging from <1% to 40%) and the presence of autoantibodies detected by the Bethesda assay or by the enzyme-linked immunosorbent assay [5]. The concomitant presence of a prolonged prothrombin time should be attributed to other causes, e.g., anticoagulants [1]. In elderly patients, morbidity and mortality associated with AHA are extremely high and, unfortunately, because of the rarity of the condition and frequently severe clinical picture at presentation, comparative studies are lacking [1]. Recent international recommendations suggest that both recombinant activated factor VII (eptacog alfa) and activated prothrombin complex concentrate, named bypassing agents, and recombinant porcine factor VIII (Susoctocog alfa), a substitutive therapy, can be administered as the first-line hemostatic treatment options [1]. Because of its lower efficacy and the risk of fluid overload, heart failure and severe hyponatremia, especially in elderly patients, the use of human factor VIII concentrates associated with desmopressin is limited to those cases where therapy with bypassing agents is not available [1,6]. Here, we report a clinical case of AHA in an elderly patient presenting with diffuse subcutaneous hematomas and anemia, successfully treated with Susoctocog alfa.

## 2. Case Presentation

**Figure 1:**
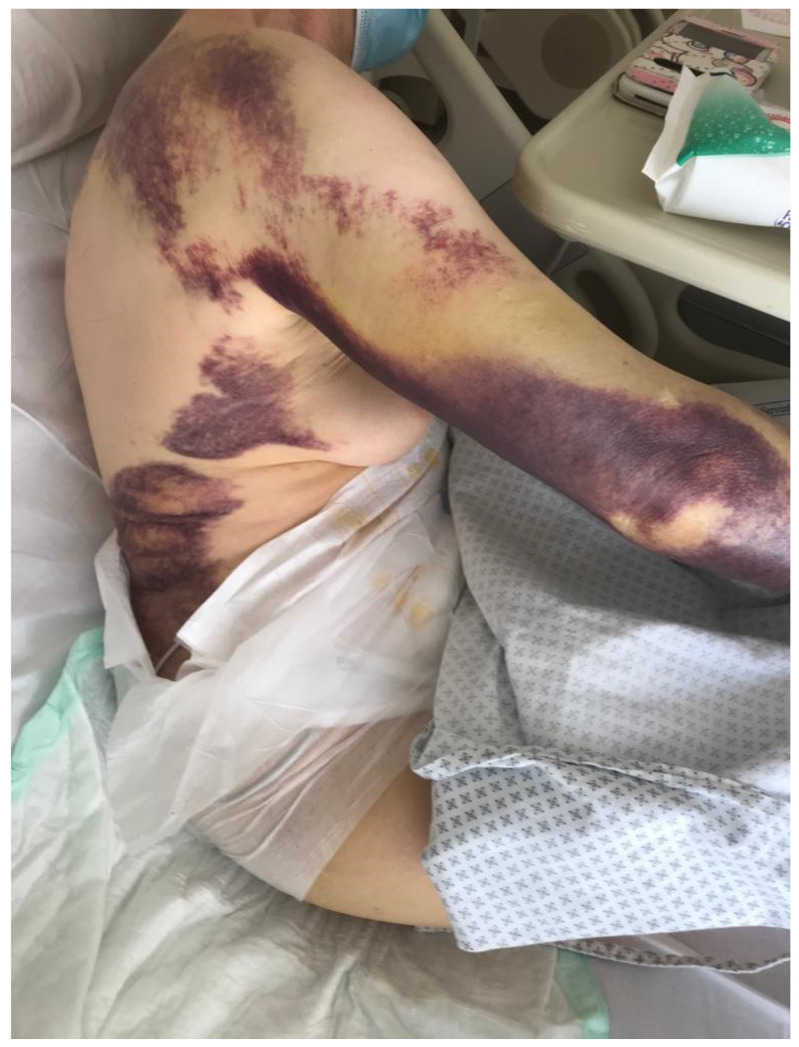
Multiple subcutaneous hematomas present at the physical examination.

**Figure 2:**
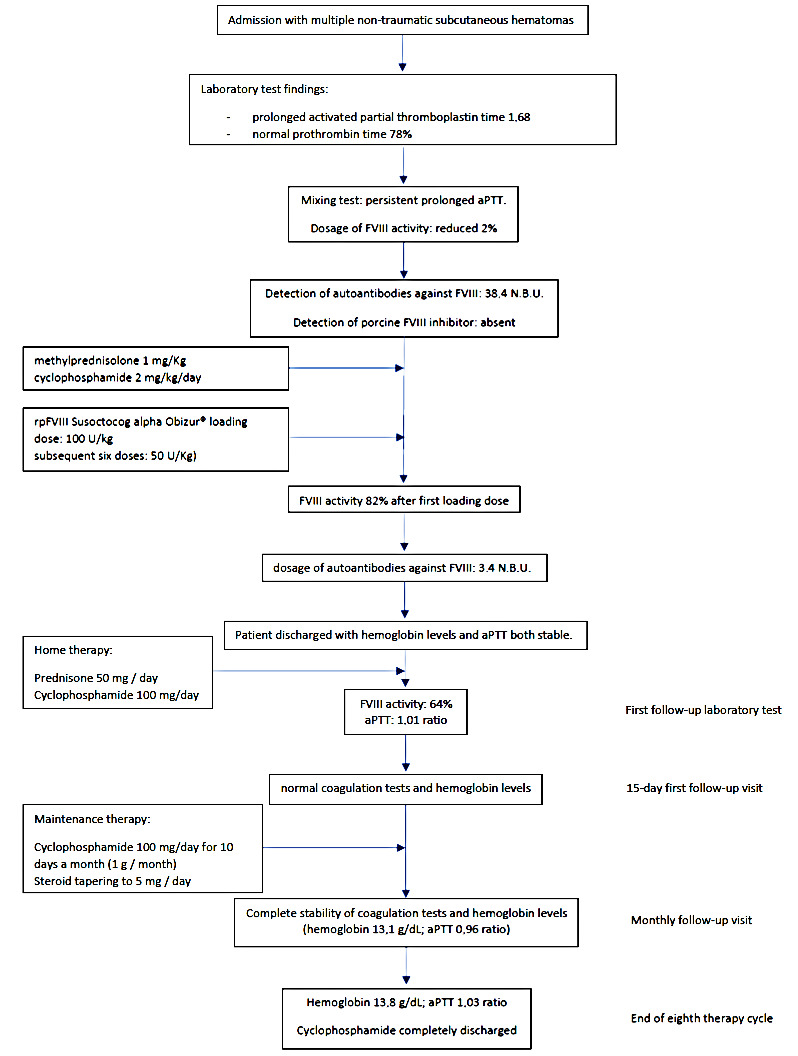
Flowchart of the case report’s clinical course.

A 79-year-old woman was admitted to the hospital for lipothymia and the presence of non-traumatic subcutaneous hematomas in the four limbs. The medical history showed ischemic heart disease with a six-month-earlier, non-ST-segment elevation myocardial infarction treated with percutaneous angioplasty and stenting, monoclonal gammopathy, hypertension and dislipidemia. The ongoing therapy was Nebivolol 5 mg, acetyl salicylic acid 75 mg, Perindopril 10 mg, Rosuvastatin 20 mg and Furosemide 25 mg bid. Ticagrelor 90 mg was stopped ten days earlier in agreement with the cardiologic follow-up. The physical examination showed hypotension and multiple subcutaneous hematomas at various stages, which were spread to the thighs, legs and arms (Figure 1). The laboratory tests showed worsening moderate-to-severe normocytic normochromic anemia, stage-I acute kidney injury and prolonged activated partial thromboplastin time (aPTT) with normal prothrombin time (PT), 1.68 ratio and 78%, respectively. The serum protein electrophoresis showed the known presence of a minor band in the gamma region (estimated 6%). No Bence Jones proteinuria was found. The patient was treated with multiple red blood cell concentrates and normal saline rehydration. The mixing test showed a persistent, prolonged aPTT thus suggesting the presence of factor inhibitors. Further investigation showed a reduced FVIII activity (2%) with normal activity of FIX, FXI and FXII. According to the international recommendations published in 2020 by Tiede et al. [1], acquired hemophilia A was diagnosed and confirmed with the positive finding of autoantibodies against FVIII detected by the Nijmegen-modified Bethesda assay (38.4 N.B.U.). As recommended, methylprednisolone 1 mg/Kg and oral cyclophosphamide 2 mg/kg/day were started as soon as possible. Accordingly, the presence of porcine FVIII inhibitor was excluded. In order to limit the thromboembolic risk, especially related to the recent cardiac procedure, rpFVIII Susoctocog alpha Obizur^®^ was used as the first-line therapy (loading dose: 100 U/kg; subsequent six doses: 50 U/Kg). FVIII levels were measured immediately before and after the first loading dose, and the results were 5% and 82%, respectively. In a few days, no other subcutaneous hematomas or internal bleeding were noticed, and supportive transfusions were not needed anymore. A further dosage of autoantibodies against FVIII showed a significant reduction (3.4 N.B.U.). When hemoglobin levels and aPTT were persistently stable (12.6 g/dL and 1.06 ratio, respectively), the patient was discharged with an instruction to continue oral prednisone and cyclophosphamide for four weeks. At the first laboratory follow-up visit, FVIII and aPTT were 64% and 1.01 ratio, respectively. At the first follow-up visit, the coagulation tests as well as hemoglobin levels were normal. The patient continued therapy with cyclophosphamide 100 mg and tapered the steroid therapy. At the end of the eighth therapy cycle, given the complete stability of the coagulation tests and hemoglobin levels, cyclophosphamide was completely discharged. A further follow-up visit showed stability of all parameters. Figure 2 summarizes the case report.

## 3. Discussion and Literature Review

In patients with AHA, bleeding is the first sign and the trigger for diagnosis in 89% of cases [2]. Because of the high risk of bleeding and the significant bleed-related morbidity, irrespective of the bleeding phenotype of the patient, immunosuppressive therapy (IST) should be started as soon as possible simultaneously with the hemostatic treatment [7]. From the first guidelines back in 2009 [6] to the most recent one in 2020 [1], IST has changed significantly; the updated recommendations suggest the use of corticosteroids alone for 3–4 weeks in those patients with FVIII ≥ 1% and inhibitor ≤ 20 BU and the use of a 3–4 week combination therapy (corticosteroid and cytotoxic agent or rituximab) when FVIII < 1% or inhibitor titer > 20 BU [2]. In contrast with congenital hemophilia, because of the high risk of bleeding and the lack of protection from new bleeding until FVIII is over 50%, the hemostatic treatment should be started simultaneously with IST. The choice of the most appropriate hemostatic treatment is usually based on the availability of agents, the anti-porcine titer, costs, monitoring requirements, personal experience and the risk of thrombotic events and safety profile [1,8]. In congenital hemophilia, both plasma-derived and human recombinant FVIII replacement therapy showed efficacy in restoring FVIII activity. Autoantibody (inhibitor) development, which emerges in approximately 30% of patients and shows type-I kinetics, represents the most serious complication associated with these treatments because bleeding episodes no longer respond to standard FVIII replacement [9]. In contrast, in AHA, the interaction between FVIII and inhibitors has type-II kinetics, so human FVIII concentrate showed significantly lower efficacy rates than bypassing agents, and its use has been limited to patients with higher initial FVIII levels and lower inhibitor titers. The porcine plasma-derived FVIII concentrate together with the more recent recombinant porcine factor VIII (Susoctocog alpha Obizur^®^) showed a low rate of anti-FVIII autoantibody cross-reactivity while maintaining high efficacy [1].

Treatment with both recombinant activated factor VII, activated prothrombin complex concentrate and recombinant porcine factor VIII is associated with a certain risk of arterial and/or venous thrombotic events, especially in elderly patients with risk factors (e.g., cardiovascular disease), recent thromboembolic events and immobility due to the present bleeding [1]. The administration of recombinant porcine factor VIII, both at an initial dose of 200 U/kg with subsequent doses assigned by clinical response and FVIII activity [10] and at a reduced dosage (100 U/Kg of loading dose and subsequent doses at 50 U/Kg), has proven to maintain excellent hemostatic efficacy with a possibly lower risk of thromboembolic events in small case series [11,12,13,14] and in the pivotal phase II/III trial [11,15] when compared with other bypassing agents. Specifically, recombinant factor VIIa (eptacog alfa activated) showed a thromboembolic risk of 0–5% and 2.9% in a systematic review [16] and in the EACH2 registry [17], respectively; the thromboembolic risk related to the administration of activated prothrombin complex concentrate was found to be 4.8% in the EACH2 registry [16]. In our case report, the decision to administer recombinant porcine factor VIII was made after a careful evaluation of the thrombotic risk, which was extremely high in our patient, and the efficacy of each recommended therapy. Specifically, recombinant porcine factor VIII (Susoctocog alpha Obizur^®^) showed high hemostatic efficacy in the available literature and presented a lower thromboembolic risk. Moreover, the loading dose and subsequent doses, even administered at a lower dosage, showed efficacy in hemostasis without thromboembolic events despite the patient’s age, comorbidity and recent myocardial infarction treated with stenting. In addition, as described in the international recommendations, the efficacy and safety of Susoctocog alfa can be monitored by measuring the FVIII activity using readily available standard FVIII assays, thus guiding the adequate dosing [1]. This result may bring us to consider recombinant porcine factor VIII as the first-line therapy in future patients with AHA and a high thomboembolic risk.

## 4. Conclusions

Although it is a rare condition, clinicians should be aware of AHA as a potential cause of bleeding in patients admitted to the hospital for hemorrhage and hematomas, especially in elderly patients with comorbidities and often ongoing antiplatelet and/or anticoagulant treatment that may mimic and mask the characteristics of the disease. Moreover, the choice of the most adequate hemostatic approach is challenging; clinicians should correctly balance the bleeding risk and the thromboembolic risk in the context of the clinical situation of the patient. With our report, we confirm the absence of thrombotic events related to the use of Susoctocog alfa as the first-line treatment option in patients diagnosed with acquired hemophilia A.

## Data Availability

Not applicable.
